# Feature-Based Sentimental Analysis on Public Attention towards COVID-19 Using CUDA-SADBM Classification Model

**DOI:** 10.3390/s22010080

**Published:** 2021-12-23

**Authors:** Siva Kumar Pathuri, N. Anbazhagan, Gyanendra Prasad Joshi, Jinsang You

**Affiliations:** 1 Department of CSE, KLEF, Vaddeswaram, Guntur District, Guntur 522502, Andhra Pradesh, India; spathuri@kluniversity.in; 2 Department of Mathematics, Alagappa University, Karaikudi 630003, Tamil Nadu, India; anbazhagann@alagappauniversity.ac.in; 3 Department of Computer Science and Engineering, Sejong University, Seoul 05006, Korea; 4 Seculayer Company, Ltd., Seoul 04784, Korea

**Keywords:** GPU, reviews, SADBM, parallel processing, CUDA

## Abstract

The COVID-19 pandemic has spread to almost all countries of the World and affected people both mentally and economically. The primary motivation of this research is to construct a model that takes reviews or evaluations from several people who are affected with COVID-19. As the number of cases has accelerated day by day, people are becoming panicked and concerned about their health. A good model may be helpful to provide accurate statistics in interpreting the actual records about the pandemic. In the proposed work, for sentimental analysis, a unique classifier named the Sentimental DataBase Miner algorithm (SADBM) is used to categorize the opinions and parallel processing, and is applied on the data collected from various online social media websites like Twitter, Facebook, and Linkedin. The accuracy of the proposed model is validated with trained data and compared with basic classifiers, such as logistic regression and decision tree. The proposed algorithm is executed on CPU as well as GPU and calculated the acceleration ratio of the model. The results show that the proposed model provides the best accuracy compared with the other two models, i.e., 96% (GPU).

## 1. Introduction

Human expertise and emotions are two crucial factors of human nature. Artificial intelligence researches study and understand the underlying behavioral mechanisms of humans. Computer systems and applications can perceive human components like feelings, which provides human behavior, and verbal exchange capabilities, which play an important role in human–computer interaction (HCI). Picard, who created the concept of emotional computing [1], gave the relevance of emotion in HCI and was applied for computer technology, cognitive technology, and other psychological studies about human emotion. An emotional calculation enables computers to recognize the emotional state and behavior, and build systems and apps that may track, examine, understand, and exploit users’ emotional states to bridge the gap between emotional human beings and computers [2]. As of 2019, there has been an outbreak of Coronavirus Disease (COVID-19). The World Health Organization (WHO) declared that the virus had spread to every country. WHO encourages people not to panic by taking preventative steps, like wearing masks, taking steam often, sanitizing their surroundings, and maintaining social distance. The new wave is getting increasingly difficult to manage because the Government has temporarily stopped lockdowns in some areas, and people started moving freely, including hotels, schools, travel, and entertainment. In this circumstance, there is a need for a model that forecasts the precise condition regarding the pandemic and alerts individuals about the importance of not getting infected by this deadly virus. Opinion or emotion process or sentiment analysis (SA) is a tool that uses machine learning to define emotion [3] as subjective data. Machine Language is one of the most prominent disciplines in natural language processing (NLP) and allows many individuals to see and assess opinions. It is a useful resource for all sorts of individuals, much like Facebook [4] and Twitter, and other social media sites. These are popular social networking sites where people can freely exchange their ideas, opinions, and feelings anytime, anywhere. One of the fundamental issues of Natural Language Processing (NLP) is analyzing an emotional representation or interpretation of an opinion [5]. For example, based on the ideas and feedback received from many individuals, entrepreneurs can understand how to sustain themselves in the market and make a vision for the future [6]. As a result, these individuals seek to develop their market or website by providing the highest quality information. Twitter and Kaggle are among those from which the data set is gathered.

## 2. Literature Survey

Online social platforms [7], such as Facebook, Linked In, and Twitter, are the various sources used for collecting datasets to perform sentiment analysis. The Twitter dataset provides much information on a person’s attitude, sentiments, and opinions. At the beginning of the epidemic, many researchers started analyzing data or reviews provided by various persons affected with COVID-19 [8] using various machine learning approaches. Worldwide investigations have been conducted to determine why this virus is spreading so quickly from person to person and why the number of deaths has increased. As a result, people began to worry about the virus’s spread as well as its consequences. To analyze this, we need a model which produces better accuracy by predicting the data collected from the Kaggle repository. This dataset consists of various opinions or reviews posted by the people affected by this deadliest coronavirus disease. The intelligence analysis method of text or opinion is used to extract the characteristics of sentiment analysis. The coronavirus pandemic has caused a massive disturbance to public welfare, health, etc. worldwide [9], which has affected people both financially and mentally. After several investigations, scientists determined that this virus is spreading due to a lack of immunity power, and also the individuals who were suffering from chronic disease [10], and by the end of 2020, this number may reach 132 million.

Aside from the viral outbreak, many bogus statements and criticisms of the movement continue to circulate on social media addressing health information and public attention. As part of this paper, we used a dataset of Indians who had been infected with this virus. A new quantitative classification model for text data analysis has been built to achieve the objective. For this, we used some existing machine learning (ML) algorithms [11], like Naive Bayes (NB) and Support Vector Machine (SVM), as base classifiers [12] and compared them with the proposed model. Therefore, from the obtained results, it is concluded that the proposed algorithm gave a better result when compared with the other two. The proposed model is based on cascading of both bagging and boosting techniques. Bagging: Bagging or batch processing is a procedure that can include the results of multiple models (such as any decision tree) to produce meaningful results. Bootstrapping is one of the sampling techniques used to generate analytic subgroups from the original dataset. The size of the subset is the same as the size of the original dataset. Multiple subsets are generated from the original dataset by selecting alternate cases. A baseline model (weak model) was built for each of these subgroups. Moreover, these models work together in parallel. All estimates are mixed to produce the final prediction.

Boosting: Loading or Boosting is a sequential operation where the next model replaces the errors in the previous model. This article deals with a hybrid algorithm that combines both bagging and boosting and is executed on GPU. Due to its hybrid nature [13], this algorithm produces a classifier that removes bias at each learning level while adjusting for overfitting. While working with the proposed algorithm for achieving better results, a few questions were encountered, which were to be appropriately answered. Q1: What are the popular keywords in Indian reviews? Q2: How do these reviews affect the public health system? Q3: How does the proposed algorithm help analyze people’s emotions or feelings [14]? Q4: How is the proposed classification model better than the other two traditional existing models.

Guo et al. proposed a novel approach by applying logistic regression and a linear discriminant model, which produced the best accuracy compared with an existing model. Guven et al. [15]. proposed an algorithm named n-stage LDA, which was the most successful algorithm for predicting emotional analysis [16]. The proposed algorithm best suits analyzing the market data using TSS (Twitter Sentiment Score), which predicts future stock market prices. Kaur et al. proposed the comparison of three basic classifiers by considering the reviews posted by the people on Twitter regarding the coronavirus in which it has been executed on CPU [17]. Chandra and Krishna proposed an algorithm for multi-labeled sentiment classification like LSTM [18] with global vector embedding and the BERT model for predicting the tweets containing more than one opinion that can be expressed at once. This paper shows that chatbots were more likely to be used for negative connections with the situation. During this pandemic, many social websites posted various reviews with some false information that made many people afraid and panic. Many gossips and false articles about the coronavirus are circulating in the media, and it is becoming challenging to distinguish them. Therefore, to fill the research gaps in Tweet approval, we proposed an enhanced model which increases the accuracy by executing it on both CPU and GPU and comparing the results with existing classifiers. The results show that the proposed model gives less acceleration ratio and higher accuracy than the other models when running on both CPU and GPU [19]. Table 1 presents the comparison of algorithms used and their accuracy.

## 3. Methodology

**Figure 1:**
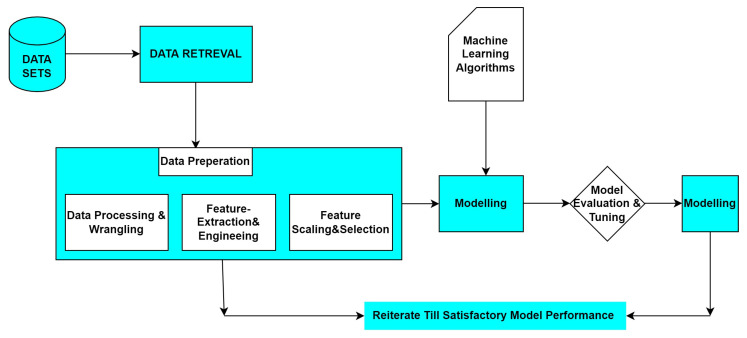
Architecture of the model.

This section examines how to utilize the API to collect information and the preliminary procedures taken for the research. The reviews or data range from December 2019 to December 2020. These reviews are from the Kaggle repository and were conducted by the state government of India. The second part of the information set consists of a series of daily individual assessments. Figure 1 depicts the model’s architecture.

Illegal actions on the Internet became a worldwide issue. Although false news is not a primary issue, it has become a serious issue. The scenario with the coronavirus epidemic demonstrates the significance of new information which has to be collected daily. Obviously, spreading misleading information impacts people’s opinions and changes the viability of Government-approved countermeasures. Inaccurate data on social media may cause tension among coronavirus patients. Twitter is a popular online media platform and microblog environment where people can post and suggest messages called “tweets”. Approximately 500 million tweets appear on Twitter every day [20], and 200 billion tweets appear every year because it has become an important information center for online media to discuss social, global, and societal problems. Chakraborty K. et al. described in 2020 that most tweets about the coronavirus are speculation, but people are usually busy spreading it. When evaluating word repetitions in tweets, they found that the tweets sounded negative and only a few positive words. Therefore, people started worrying and getting panicked by reading these reviews or tweets; thus, in this situation, it is necessary to propose a predictive model that predicts the polarity of the tweets and gives better results to make people aware of COVID-19 mentally and physically. Furthermore, make them take necessary precautions without getting affected by this deadliest virus.

### Dataset

The records or dataset used in this paper includes the statistics of Indian tweets from the Twitter website (online) taken during the Coronavirus lockdown implemented across the country. The informational index comprising 90,000 tweets has been extracted from kaggle.com, accessed on 10 October 2021, and it includes clean tweets on particular words like coronavirus, COVID, lockdown, etc. The dataset [21] is comprising of eliminated tweets from the original dataset taken into consideration for examination.

**Figure 2:**
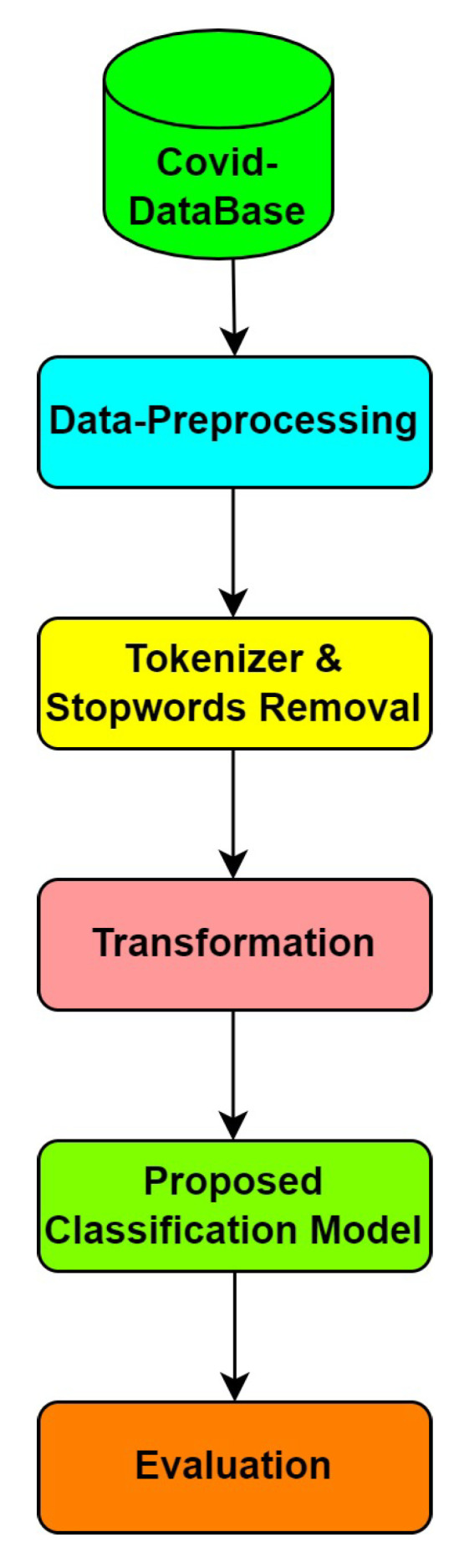
The work flow of the proposed model.

Figure 2 shows how we allocated the collected comments or tweets to a certain length and mainly divide the information according to the tags [22] like panic, frustration, anger, and funny. These values are mapped to 4 class labels, called 1, 2, 3, and 4, where (1 = panic, 2 = frustration, 3 = irritated, 4 = interesting). In the data set, the ratings are rated as positive, negative, or neutral, some of them are like “good” and “excellent” and some bad sentences like “dying”, “killing”, “panic”, “sadness”, etc. Based on emotions that are conveyed through the use of unique characters in the dataset, we have implemented the process diagram, as shown in Figure 2. It shows the percentage of sentences and emotions in the dataset (NLP). Generally, NLP uses various methods to extract text content to make information easier to retrieve. Such as noise reduction, prevention or buzzwords, etc. Therefore, the suggested model gives better results when compared with other classifiers.

## 4. Compute Unified Device Architecture (CUDA) and Programming

**Figure 3:**
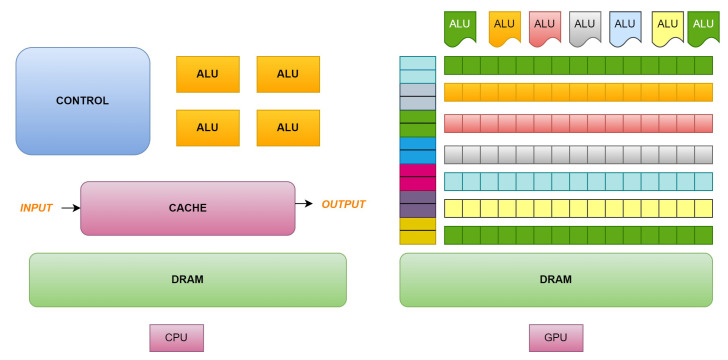
General architecture of GPU versus CPU.

Compute Unified Device Architecture (CUDA) was designed for general computing using NVIDIA’s graphics processing unit, which acts like parallel programming (GPU) [23]. CUDA helps developers speed up computer-intensive programs by using the capacity of the GPU to perform parallel calculations. In 2003, some set of people proposed Compute Unified Device Architecture (CUDA), an extension of C with parallel processing of data. Compute Unified Device Architecture (CUDA) has various applications used to boost the program’s speed by dividing the instruction into various threads. It is known as computing unified device architecture, which contains predefined library functions or methods for boosting the processing speed. It works on the principle called parallel computing to increase speed as well as performance. The second principle for why we chose parallel computing is multicore processing. In parallel processing, the code is divided into several threads in which each thread works effectively, providing a better acceleration ratio. CUDA on NVIDIA graphics card, which runs on a peculiar compiler called nvcc. This nvcc gives instructions for both the host and GPU, which in turn communicate the data between them. Figure 3 shows how an architecture of GPU and CPU will be like.

In parallel processing, it is impossible to access memory directly, and also CPU cannot access the GPU memory, so in that case, we require data to copy explicitly using CUDA predefine library functions or methods [24]. In the CUDA hierarchy, the code is divided into threads, which in turn forms a block of thread, which in turn these blocks together forms grids like Grid0, Grid1...., with corresponding per-thread private, per-block shared, and per-application global memory spaces. Basically, the working of GPU is quite different when compared with CPU. Modern GPUs are very efficient in performing various applications like image processing, machine learning, etc. These applications are embedded as firmware on the mainboard itself so that the processing time could take less when compared with the CPU. CUDA is a parallel programming model along with some instructions set. Therefore, in this paper, we used CUDA 2.1 with C language for obtaining the results and the CPU used is Intel i7 5th generation with 2.25 GHz speed processor for comparing the results both on GPU as well as CPU which is discussed in Performance Evaluation Measures section.Generally CUDA contains CPU code with atleast one kernel, i.e., void returning module need to be implemented by GPU. This architecture contains some predefined keywords for ex __global__ is a kernel function called by CPU [25], and we executed on our GPU.and the __device__ is used for calling the threads which are executing on GPU.The __host__ is a keyword used for calling function only by CPU.While working on parallel computing we can combine both __host__ and __device__ [26].

### GF108 Architecture

The GF108 is the core of GT 525 m which is combined or connected with GF100 core which in turn forms Geforce GTX 480 M which provides 128 bit memory bus with 96 shaders for SSD VRAM3 [27]. NVIDIA’s GF108 uses Fermi architecture made up with 40 nm process production at TSMC. Which contain a die with size approximately 116 mm${}^{2}$ along with transistors of about 585 million.To perform GPU computing Open CL along with CUDA 2.1 is used which contains 96 shaders with 16 texture mapping and 8ROP’s.Unlike GF108 cores will be considerably adjusted when compared with GF100. In GF108 the shaders will be in the form 3 × 16 instead of 2 × 16 along with textures 8 instead of 4 along with special unit called SFU.Nvidia uses special super scalar processor which forms parallelism called instruction level parallelism on a single processor so that the utilization of shaders per SM will be more effective.

## 5. Proposed Method

**Figure 4:**
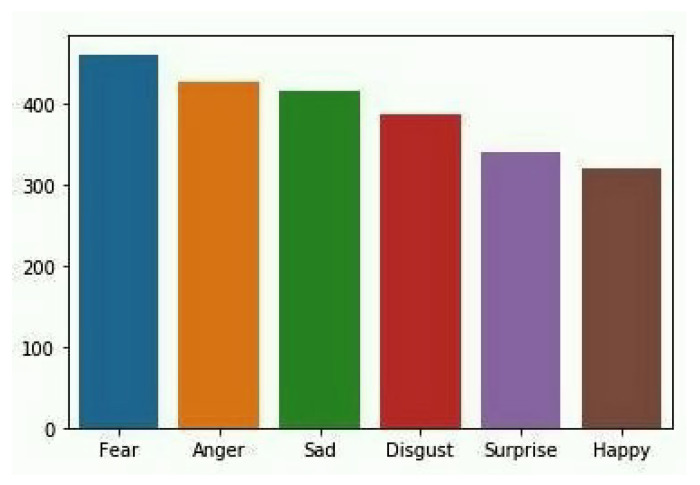
Review count based on polarity.

**Figure 5:**
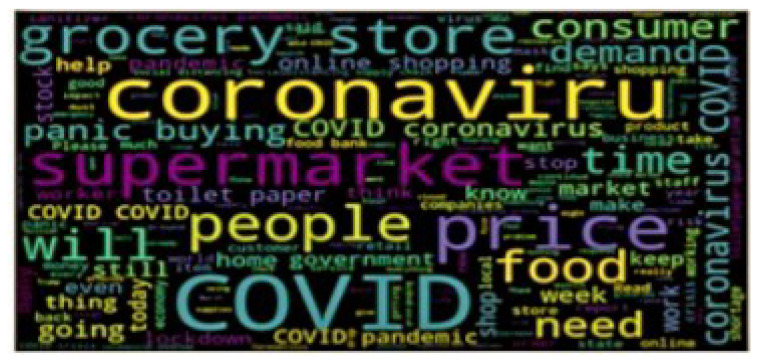
Word cloud of a review.

Logistic Regression: It is one of the ML algorithms which is used for the classification of data. This algorithm gives the possible outcome of the model by using a logistic function [28]. The main advantage of using this model is how various independent variables affect a single outcome variable. It is commonly used when the taken dataset has only two outputs, i.e., 0 or 1. It is used when the data is categorical. Figure 4 shows the count of reviews, and Figure 5 shows the word cloud of a review.

### 5.1. Making Predictions with Logistic Regression

Using an LR model, we make predictions that are as simple as giving values to the logistic regression equation [29] and calculating the result. Let us use a simple example to make it easier to understand. Suppose we have a model which has to predict whether a review given by a person is positive or negative [30]. About COVID-19 (completely fictitious), based on the review, we calculate the polarity of that review by considering the coefficients of b0 = −0.05 and b1 = 0.05. With the above equation, we can calculate the probability that a person’s formal P (human|review ≤0.05) is considered negative if P (human|review ≥ 0.05) is considered positive.

In general, we can use the probabilities directly as the probabilities are of binary class value, for example,

0 if p (review) < 0.05

1 if p (review) ≥ 0.5

**Figure 6:**
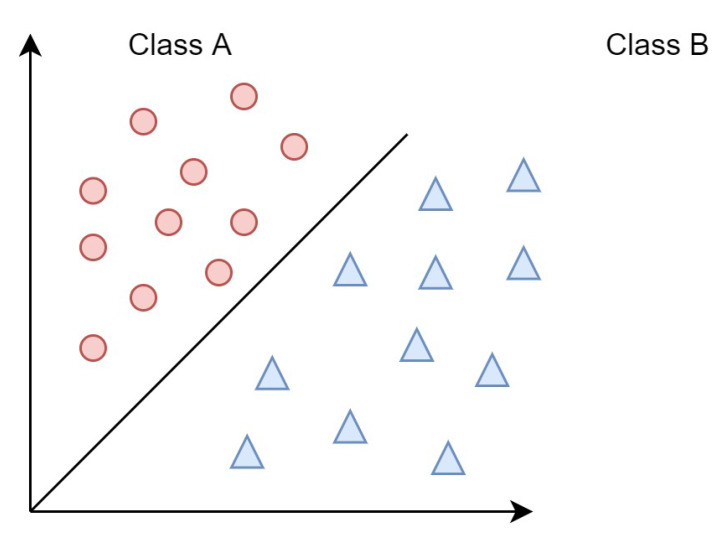
Data classification.

Finally, the machine learning algorithm is used for predictive modeling. The LR model provides better predictions than just interpreting the result. Figure 6 shows how the data is classified, and as long as the model is robust and works well, we can break some assumptions. (1) Binary output. (2) Elimination of noise. (3) Use of the Gaussian distribution. (4) Elimination of correlated I/P.

### 5.2. Decision Tree

**Figure 7:**
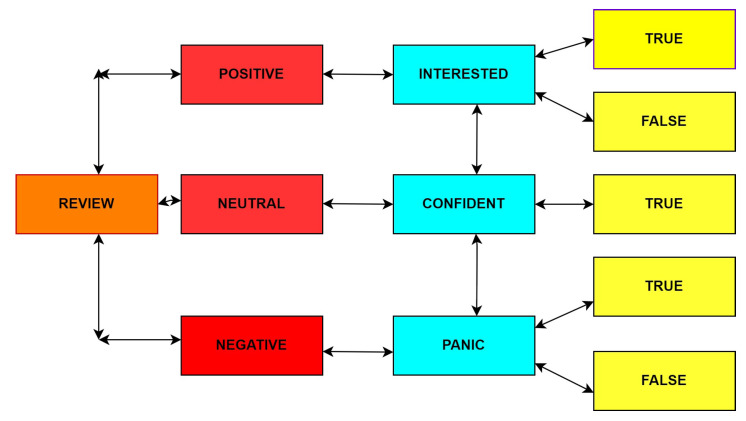
Decision tree of a review.

In general, decision trees may be used to solve classification or regression models that predict the output based on feature-based split [31]. A decision tree contains the following terms: root node, split, decision node, leaf node, and pruning. Basically, it starts with the root and divides the dataset into smaller and smaller subsets while gradually developing a coherent decision tree that ends with a decision provided by leaf nodes [32]. A decision node, such as Review, may contain two or more branches: positive, negative, and neutral. Leaf nodes, such as interested, confident, and panic, represent categories or solutions. The node with maximum entropy that will be considered the best predictor in the tree is called the root node. A decision tree can handle categorical and numerical data. Figure 7 shows the decision tree of a review. The base algorithm to implement a decision tree is ID3, which follows the top-down approach without backtracking. It uses a strategy called entropy and Info gain. In which the ZeroR model contains no predictor, and the OneR model includes the single best predictor.

#### 5.2.1. Entropy

**Figure 8:**
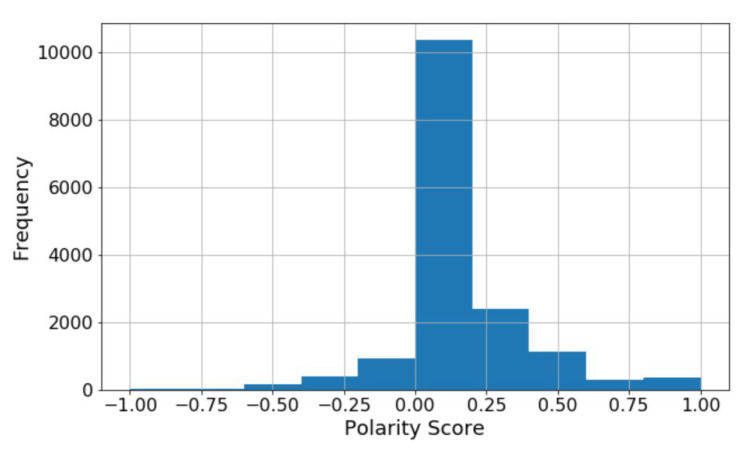
Polarity vs. frequency.

**Figure 9:**
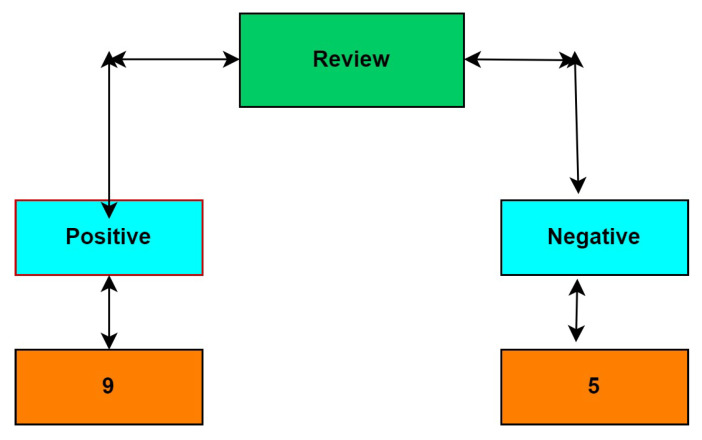
Finding positive and negative words for Sample review.

In the decision tree, we follow the top-down approach, i.e., from the root node, and divides the data into subsets consisting of several instances with homogenous values. Entropy has to be calculated for the given sample input in the ID3 algorithm. If the O/P is single, then entropy is considered as 0. It is considered as 1 if the sample I/P is divided equally. The following are the figures used to show how we can calculate the entropy of a review shown in Equation (1), and how the review’s polarity along with its frequency represented in histogram which is shown in Figure 8, and also how the positive and negative words for a sample review can be calculated shown in Figure 9. And finally in Equation (2) shows how to calculate entropy for 2-attributes.

(1) $$Entropy\left(P\right)=-\sum_{i=1}^{N}P_{i}log_{2}P_{i}$$

To construct a decision tree, we may construct two frequency tables. 1. Frequency table to find entropy with single attribute:

Then Entropy is calculated as Entropy (Review) = Entropy (5,9) = Entropy (0.36,0.64) = 0.94 2. Frequency table to find entropy with 2 attributes: Entropy (Label, Review) = 0.71

(2) $$Entropy\left(T,X\right)=\sum_{C=1}^{X}P\left(C\right)E\left(C\right)$$

#### 5.2.2. Information Gain

The use of information gain is to reduce entropy caused by the partition of the examples based on the specified attribute after it is splitted.To construct any decision tree, find the best attribute which gives us the best info gain. To find the information, gain we have certain steps to follow.

- Find the entropy of the target attribute.
- Entropy of every branch is calculated to find the best split.
- Select attribute with high info gain and recursively repeat the same for all the remaining branches.
- If entropy is zero, then consider all of them as a leaf node; else, continue splitting.
- Repeat all the above steps until data is classified.

### 5.3. Proposed Compute Unified Device Architecture (CUDA) Sentimental Analysis Database Miner Classifier

This article aims to predict the model’s accuracy when executed on GPU, with less processing time than CPU. Furthermore, with the increase in the acceleration ratio, the model’s accuracy gets increased compared with the existing classifiers. Data mining is an approach for collecting data from a wide range of real-world data. Our methodologies, such as rating, categorizing, etc., are long-established. We used some typical ratings in the suggested technique. It comprises numerous characteristics in the COVID-19 data from the Kaggle repository. The dataset includes numerical and categorical details, such as viewing, location, timing, summary text, etc. It is based on the notion of IF-THEN. The suggested SADBM works in two stages: In Algorithm 1, a decision tree based on extracting the functionalities utilized to determine the polarity of each review posted will be generated. From the obtained confusion matrix, some standard measures for predicting the accuracy like precision, recall, F-score of the model in Algorithm 2 are calculated in the second phase [33].
**Algorithm 1: **Decision Tree Building Algorithm
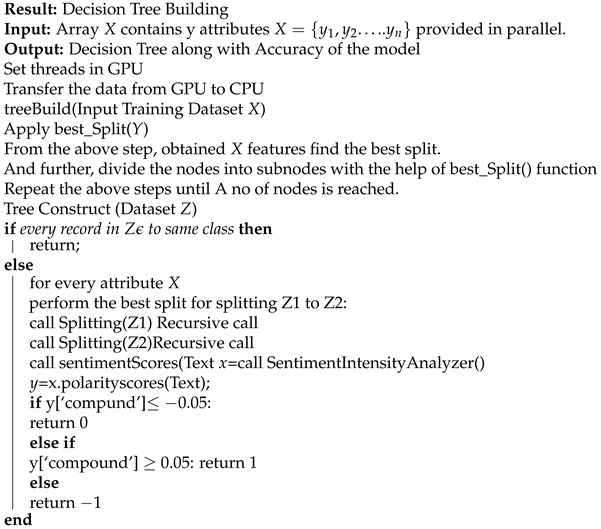

**Algorithm 2: **Accuracy Prediction Algorithm**Call:** xgboost()M= x.xgbClassifier()m.fit(X_train,Y_train)**Call:** ConstructTree()c= confusion_matrix (Y_test,Y_pred)Calculate: Accuracy = Number of correct predictions/total predictionsReturn accuracy**Stop**

## 6. Implementation and Results

**Figure 10:**
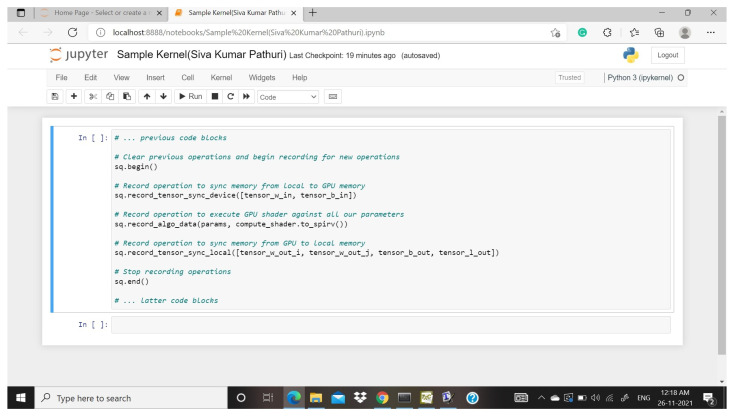
Sample kernel code.

The classifiers, as mentioned earlier, like Decision Tree, SADBM, and Logistic Regression, are executed with the help of GPU, i.e., CUDA programming, where all the algorithms gave less processing time compared with CPU processing time. In this paper, the SADBM and the other two algorithms were executed on GPU with the help of CUDA [34], and then we calculated the acceleration ratio of every algorithm, in which the proposed model gave the best accuracy compared with the other two. Table 2 shows the number of threads and the time taken to process those threads. Figure 10 tells that how local CPU/host resources bind with the shader and also how data mapping is done in the GPU memory into the local Tensor vector.

As the number of threads getting increased, the time taken to process the data is getting decreased, which is why GPU computing is very effective compared to CPU. Table 3 shows the acceleration ratio to classify the records (data) using SADBM.

### 6.1. Acceleration Ratio

To calculate the performance of GPU we have to find out the acceleration ratio of GPU, i.e., (Speed-Up time) which is given as

(3) $$gamma=\frac{t_{CPU}}{t_{GPU}}$$

where Gamma is the total processing time taken by the CPU, divided by the total processing time taken by GPU. We calculate the CPU time by adding random value generating time, time for data sort, and time for classification. Whereas for GPU, it is calculated with inter-transfer of data, i.e., from host to device and device to host. For evaluation measures, we applied CUDA-SADBM along with Logistic regression and Decision tree. We compared the accuracy where CUDA-Sentimental Analysis Database Miner Classifier (SADBM) produced better accuracy when compared with the other two, which when executed on GPU.

### 6.2. Performance Evaluation Measures

Precision is a metric used to measure performance and data retrieval. This metric quantifies the total positive predictions given by the classifier. Mathematically it can be presented as follows:

(4) $$Precision=\frac{TP}{\left(TP+FP\right)}$$

Recall is a metric that is used to measure the performance and also for data retrieval. This type of metric quantifies the fractional part between and is manually classified, i.e., (true positive + false negative) given by the classifier. Mathematically it can be presented as follows:

(5) $$Recall=\frac{TP}{\left(TP+FN\right)}$$

F-score: It is defined as the harmonic mean between precision and recall. Mathematically F-score is given as

(6) $$F-measure=2*\frac{Precision*Recall}{Precision+Recall}$$

Finally, accuracy is given as the proportion of TP,TN,FP, and FN. Mathematically it is given as

(7) $$Accuracy=\frac{TP+TN}{\left(TP+TN+FP+FN\right)}$$

**Figure 11:**
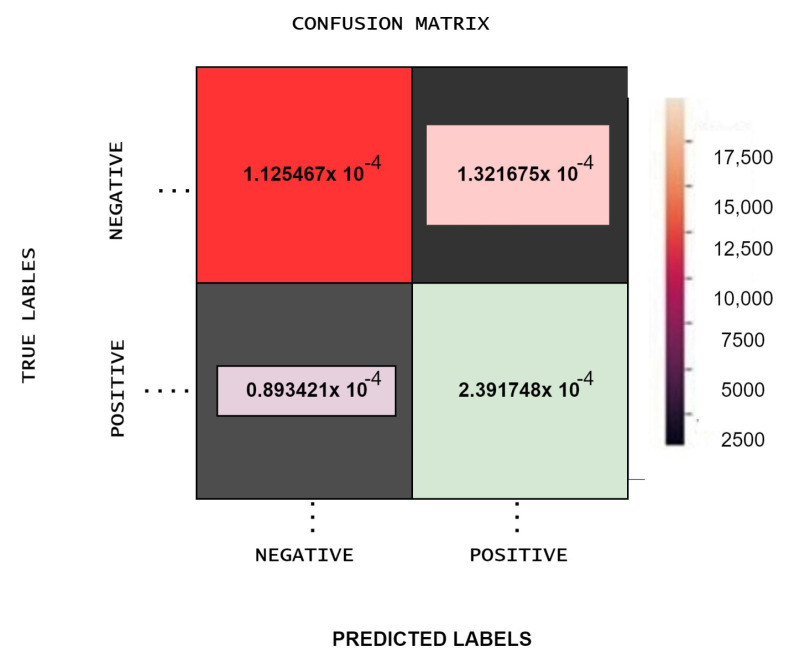
Obtained Confusion matrix for Trained Data.

**Figure 12:**
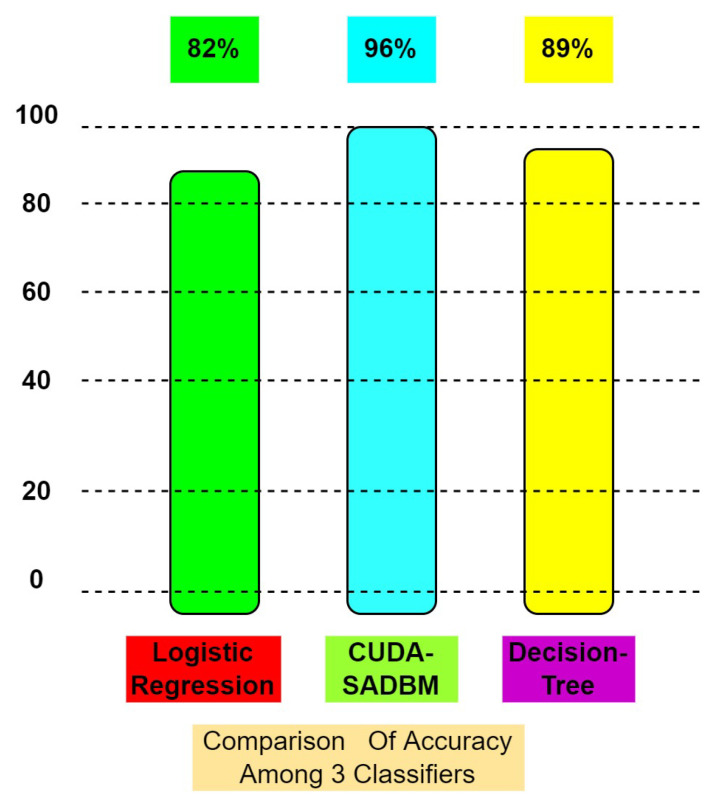
Comparison of 3 algorithms.

The result of the proposed model is evaluated with the help of a confusion matrix and then we calculated precision, recall, F-score. Finally, the obtained confusion matrix is shown in Figure 11, in which the accuracy of the proposed classifier is high with less processing time. Moreover, after executing the proposed algorithm on GPU the accuracy is compared with the other two base classifiers shown in Figure 12.

Table 4 shows the logistic classifier for multiclass classification. Table 5 and Table 6 show the decision tree classifier for multiclass classification and the Computer Unified Device Architecture-Sentimental Analysis Database Miner Classifier for multiclass classification.

## 7. Conclusions and Future Work

This article is based on the reviews or opinions given by various people across India during the COVID-19 pandemic that assist the decision-makers in helping the needy. We proposed a CUDA-SADBM classifier that can classify datasets when it is implemented in parallel computing(GPU) using a large number of attributes. We collected the reviews from kaggle repository to analyze the proposed model, and trained the data, and finally tested with three classifiers. After executing all the three classifiers on GPU the results shows that the proposed algorithm gave best accuracy when compared with logistic regression and decision trees, i.e., the acceleration over time is calculated, where GPU mining improves accuracy with less processing time. Experimental results show that the accuracy of the proposed method is 96%, whereas the accuracy rate of logistic regression is 82%, and the decision tree is 89% only. The proposed method is limited to a smaller dataset. However, if the size of the dataset is increased, the processing time increases, resulting in a decrease in accuracy. This can be mitigated by changing a few parameters. Tuning for better results in an extensive database remains for future works.

## Figures and Tables

**Table 1:** Literature survey.

Author	Algorithms Used	Feature-Selection	Data Source	Accuracy
Gualtiero Bcolombo (2015)	Graph mining	TF-IDF	Web forums(Twitter Data)	84%
Dmytro Karamshuk (2017)	Glove word vector, Conventional classification, DT	Consistency Label	Public Twitter	85%
Tong Liu (2017)	Support vector Machine (SVM)	TF-IDF, N-Grams	Historical Twitters posts	88%
Bridianne O’Dea (2015)	SVM, Logistic regression	TF-IDF wih filter and without filter and no filter, Data points	CSIRO	80%
Pete Burnap (2015)	SVM, Naïve Bayes (NB), Decision Tree (DT), Rotation forest	Lexical, Structural, Emotive, Psychological TF-IDF, N-Grams,	Web forums (Twitter Data)	75%
Benjamin. L (2016)	Logistic regression	N-Grams, Linguistic context	Kaggle	82%
Mia Johnson Vioules (2017)	NB, Sequential minimal optimization (SMO), Decision tree (J48),	NBB, Multinomial L-R, RF		80%
Scott R Braithwaite (2016)	Decision Tree (DT)	Linguistic, word count	Amazon Mechanical Turk (AMT)	76%
Munmum De Choudhury (2013)	SVM with a radial-basis function (RBF) kernel	Depression set	Crowdsourcing	86%
Ramit Sawhney (2018)	Ensemble, Linear classification	Twitter streaming API		81%
Bart Desmet (2018)	Parallel Computing	Bag of words, polarity lexicon	KAGGLE	92%
Shaoxiong Ji (2018)	SVM, random forest, gradient boost classification, XGboost	TF-IDF, semantics and syntactic, statistics, Linguistic features	Reddit and Twitter blogs	89%
Jingcheng Du (2018)	CNN binary classification	Linguistic features	Twitter streaming API	74%

**Table 2:** Number of threads versus time taken.

No of Threads	Time Taken
128	5.12
256	4.14
512	3.12
1024	2.69

**Table 3:** Acceleration ratio to classify the records using SADBM.

SADBM GPU Time	No of	No of	No of	No of	No of
	Records	Records	Records	Records	Records
	s/12,000	s/32,000	s/52,000	s/72,000	s/92,000
Classification Time	0.552	1.020	1.705	2.052	2.742
CPU-Time	0.710	1.130	1.740	2.3500	2.900
GPU-Time	0.550	1.010	1.640	2.230	2.490
Acceleration Ratio	1.296	1.118	1.064	1.054	1.1649

**Table 4:** Logistic Classifier for multiclass classification. Training accuracy Score: 0.9269020501138952. Validation accuracy Score: 0.8156025267249757.

Polarity	Precision	Recall	F-Score	Support
0	0.79	0.82	0.77	2467
1	0.884	0.81	0.84	5765
Accuracy			0.81	8232
MacroAvg	0.81	0.82	0.81	8232
WeightedAvg	0.81	0.81	0.81	8232

**Table 5:** Decision Tree Classifier for multiclass classification. Training accuracy Score: 0.9469020501138952. Validation accuracy Score: 0.8856025267249757.

Polarity	Precision	Recall	F-Score	Support
0	0.89	0.84	0.88	2899
1	0.884	0.91	0.89	5333
Accuracy			0.89	8232
MacroAvg	0.87	0.88	0.87	8232
WeightedAvg	0.89	0.89	0.89	8232

**Table 6:** CUDA-SADBM Classifier for multiclass classification. Training accuracy Score: 0.9869020501138952. Validation accuracy Score: 0.9656025267249757.

Polarity	Precision	Recall	F-Score	Support
0	0.953	0.943	0.924	2882
1	0.950	0.948	0.946	5350
Accuracy			0.96	8232
MacroAvg	0.96	0.96	0.956	8232
WeightedAvg	0.955	0.961	0.959	8232

## Data Availability

Not applicable.
